# Neuroimmunology of the Interleukins 13 and 4

**DOI:** 10.3390/brainsci6020018

**Published:** 2016-06-13

**Authors:** Simone Mori, Pamela Maher, Bruno Conti

**Affiliations:** 1 Department of Chemical Physiology, The Scripps Research Institute, 10550 North Torrey Pines Road, La Jolla, CA 92037, USA; smori@scripps.edu; 2 Cellular Neurobiology Laboratory, Salk Research Institute, La Jolla, CA 92037, USA; pmaher@salk.edu

**Keywords:** Interleukin 13, Interleukin 4, neuron, microglia, Parkinson, brain, neurodegeneration, neuroinflammation, neurotoxic, neuroprotection

## Abstract

The cytokines interleukin 13 and 4 share a common heterodimeric receptor and are important modulators of peripheral allergic reactions. Produced primarily by T-helper type 2 lymphocytes, they are typically considered as anti-inflammatory cytokines because they can downregulate the synthesis of T-helper type 1 pro-inflammatory cytokines. Their presence and role in the brain is only beginning to be investigated and the data collected so far shows that these molecules can be produced by microglial cells and possibly by neurons. Attention has so far been given to the possible role of these molecules in neurodegeneration. Both neuroprotective or neurotoxic effects have been proposed based on evidence that interleukin 13 and 4 can reduce inflammation by promoting the M2 microglia phenotype and contributing to the death of microglia M1 phenotype, or by potentiating the effects of oxidative stress on neurons during neuro-inflammation. Remarkably, the heterodimeric subunit IL-13Rα1 of their common receptor was recently demonstrated in dopaminergic neurons of the ventral tegmental area and the substantia nigra pars compacta, suggesting the possibility that both cytokines may affect the activity of these neurons regulating reward, mood, and motor coordination. In mice and man, the gene encoding for IL-13Rα1 is expressed on the X chromosome within the PARK12 region of susceptibility to Parkinson’s disease (PD). This, together with finding that IL-13Rα1 contributes to loss of dopaminergic neurons during inflammation, indicates the possibility that these cytokines may contribute to the etiology or the progression of PD.

## 1. Introduction

In this review we summarize the current body of knowledge on the role of IL-13 in the central nervous system. Although the study of this subject is in its infancy and only a limited amount of work has been done at this stage, it is likely that this will change in the near future. In fact, one of the interesting aspects of investigating the biology of IL-13 in the central nervous system (CNS) is that its canonical receptor, alpha type I (IL-13Rα1), appears to be expressed not only in glial cells during pathological conditions, but also in specific subsets of neurons in the healthy brain. Specifically, IL-13Rα1 has, so far, been found on dopaminergic neurons of the Substantia Nigra pars compacta (SNc) and the Ventral Tegmental Area (VTA) [1]. This finding indicates that its ligands, IL-13 and IL-4, could be important regulators of dopaminergic function and cell survival, and may provide a direct link between the immune system and the neurobiology of reward, addiction, or motor coordination.

## 2. What We Know about IL-13 Comes from Studies of Its Biology in the Immune System

The cytokines Interleukin 13 (IL-13) and interleukin-4 (IL-4) are two secreted proteins recognized for their role in promoting T-helper type 2 (Th2) lymphocyte-mediated allergic inflammation and atopy in the periphery [2,3,4,5,6,7,8,9,10,11,12,13,14,15,16,17]. IL-13 and IL-4 also have the ability to downregulate the synthesis of T-helper type 1 (Th1) lymphocyte pro-inflammatory cytokines: for this reason they are normally listed as anti-inflammatory interleukins [8,9,10,15,18,19,20]. Both cytokines are produced by Th2, as well as by other cell types, including eosinophils and basophils [2,5,6,9,11,12,13] and IL-13 production is also stimulated in mast cells by lipopolysaccharides (LPS) [21,22,23,24].

**Figure 1:**
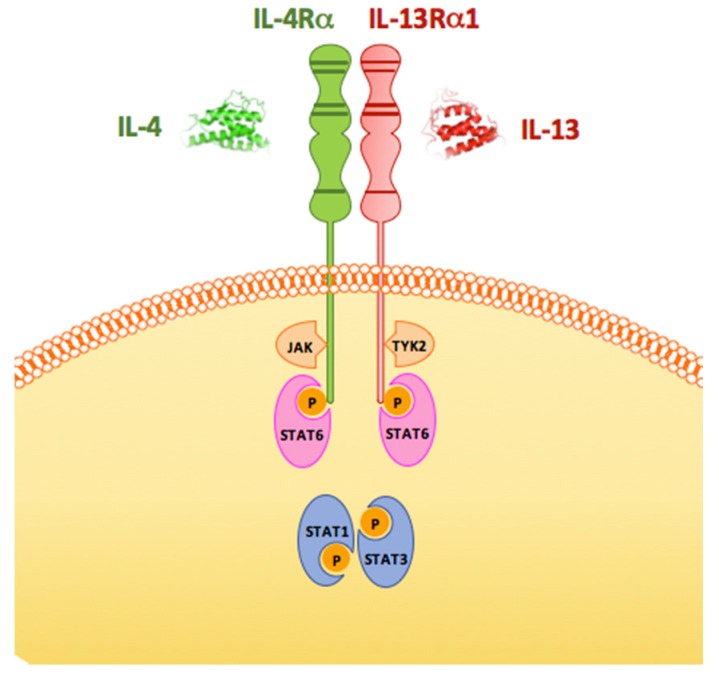
Schematic representation of the heterodimeric receptor for IL-13 and IL-4 and its signaling. Interleukins 13 (IL-13) and 4 (IL-4) can bind to the same heterodimeric receptor composed of the IL-13 Receptor alpha 1 (IL-13Rα1) and the Interleukin 4 Receptor alpha (IL-4Rα). Binding of the receptor activates the Janus kinase (JAK) and leads to phosphorylation of members of the Signal Transducer and Activator of Transcription (STAT) family. The tyrosine-protein kinase 2 (TYK2) is a member of the JAK family. See the text for more details.

IL-13 and IL-4 are often investigated together because they partially share a common receptor type: the IL-13 receptor alpha 1 chain (IL-13Rα1). IL-13Rα1 heterodimerizes with the IL-4R alpha chain (IL-4Rα) forming a complex capable of binding IL-13 or IL-4 (Figure 1) [25,26,27,28,29,30,31,32]. To date, this complex is the only known signal transducer for IL-13, while IL-4 can also signal through an IL-4Rα/gamma chain complex. A high-affinity IL-13-binding protein (IL-13Rα2) also exists and is a specific inhibitor of IL-13 signaling, likely by functioning as a decoy receptor [28,33,34,35,36]. IL-13Rα2 is not found in the healthy brain and, so far, has only been shown to be expressed in the CNS on glioblastoma cells [37] making it one of the major targets of immunotherapy. Work on IL-13Rα2 in the CNS and its role as a therapeutic target will not be discussed here and is covered by recent excellent reviews [38].

Binding of IL-13 to its cognate functional receptor allows the trans-phosphorylation of a specific tyrosine residue located in the Janus Kinase (JAK) activation segment [31,39] which promotes the kinase activity required for the phosphorylation of downstream substrates in its signaling cascades [39,40]. IL-13 activates two intracellular signaling cascades: the JAK-STAT and the insulin receptor substrate (IRS)-phosphatidylinositol 3′-kinase pathways [26,28,31]. While the IRS-phosphatidylinositol 3′-kinase pathway leads to cell proliferation, the JAK-STAT pathway induces the transcription of genes that contain the Stat6-responsive enhancer element N6-GAS located in their promoter [41,42,43]. Upon activation of IL-13Rα1, Stat1, 3, and 6 are phosphorylated and form a homodimer that migrates to the nucleus and binds to N6-GAS to drive transcription [31,42,44,45]. Reactive oxygen species (ROS) also play a role in the IL-13/IL-4 cellular transduction signaling. In intestinal epithelial cells upon IL-13Rα1 activation both the JAK-STAT pathway and Mitogen Activated Protein Kinase (MAPK) stimulate nicotinamide adenine dinucleotide phosphate oxydase to produce intracellular ROS that, in a positive feedback loop, facilitate the phosphorylation of STAT6 and ERK [46]. Moreover, IL-13/IL-4-driven ROS production has been recently shown in alternatively-activated monocytes/macrophages through activation of monoamino oxydase A (MAO-A) [44].

## 3. Expression of IL-13 and IL-4 in the CNS

As mentioned above, IL-13 and IL-4 were demonstrated to be produced peripherally. To date, there is no evidence that these two proteins, both with molecular weights in the range of 15 kDa, can cross the blood-brain barrier. However, experimental work shows, instead, their local production in the CNS. Expression of IL-13 in the rodent brain was described in microglia, where its production was enhanced by peripheral injection of LPS or the neurotoxin 1-metil-4-fenil-1,2,3,6-tetraidropiridina (MPTP) [47,48,49,50,51].

Evidence also exists that both IL-13 and IL-4 can be produced by neuronal cells of the hippocampus and the cortex in experimental models of ischemic insult [52,53]. In this context it has speculated that the production of IL-4 and IL-13, inducing alternative activation of microglia—known as the M2 state—can exert a protective effect against neuronal damage [53,54,55]. Neuronal production of IL-4 has been described lately in the noradrenergic neurons of the locus coeruleus, in which its release appears to be sensitive to behavioral stress [56]. Preliminary work in our laboratory also showed that IL-13 can be produced in neurons [57].

## 4. What Is the Role of IL-13 and IL-4 in the CNS?

Few studies have tested the effects of IL-13 and IL-4 in the CNS. Most of these have investigated a possible action on neuronal survival with some studies finding that IL-13 and/or IL-4 potentiate the effects of LPS and Interferon gamma (IFN-y), increasing oxidative damage and contributing to neuronal death [47,48,49,50,58,59,60,61]. On the other hand, other studies indicated that IL-13 and/or IL-4 could be neuroprotective either by directly reducing inflammation or by inducing the death of microglia cells that are considered to be cellular mediators of neuronal damage [47,48,49,50,59,60,61,62,63,64,65]. Notably, both IL-13 and IL-4 can potentiate LPS-induced sickness behavior when co-injected centrally with LPS, whereas only IL-4, and not IL-13, attenuates LPS-induced sickness behavior when administered several hours before LPS [47,66]. Recently, our laboratory collected evidence that IL-13 and IL-4 are not toxic when administered alone but can greatly increase the susceptibility of neurons to oxidative damage and contribute to their demise if they express IL-13Rα1 [1].

## 5. IL-13 and IL-4 in Multiple Sclerosis

Multiple sclerosis (MS) is an autoimmune disorder affecting the CNS with a relapsing-remitting time course. IL-13 seems to exert a protective role in this context, as it is believed that in the development of the disease, a crucial role is played by the imbalance between pro-inflammatory cytokines (IL-1β; TNF; INF-γ; IL-17) and anti-inflammatory cytokines (IL-4, IL-5, IL-10 and IL-13) [67,68]. IL-13 polymorphisms are associated with autoimmune diseases and also increase susceptibility to MS [69].

A study in humans with MS found that high levels of IL-13 in the cerebral spinal fluid (CSF) might exert a neuroprotective effect by enhancing Gamma Aminobuthirric Acid (GABA) over glutamate transmission [64]. Interestingly, an earlier report describes IL-4 having the same neural effect of increasing the GABA-induced inward current in neurons in a dose-dependent and reversible manner [70]. Moreover, the copolymer glatiramer acetate, an immunomodulatory drug currently used to treat MS, has shown to significantly increase the TH_2_- lymphocyte production of IL-13 in patients [71].

Consistently, using the mouse experimental model of MS, experimental autoimmune encephalomyelitis (EAE), Cash and colleagues showed that IL-13 exerts its anti-inflammatory action by inactivating macrophages and reducing oxidative stress [72]. In the same model, an increase in circulating and spleen IL-13 prevented axonal injury [73] alone or in synergy with IL-4 [74], whereas IL-13 reduction was associated with loss of protection [75].

Sex difference can play a role in affecting the role of IL-13 in the MS model. While autoimmune diseases, including MS, are more common in women [76], the incidence and severity of EAE in mice, null for IL-13, was lower in females compared to males, suggesting the possibility that the contribution of IL-13 to EAE/MS may be gender specific [77]. To this end, it is interesting to note that the expression of IL-13 mRNA can be decreased by estrogen in a mouse model of inflammatory intestine disease [78] and that the gene encoding for IL-13Rα1 is located on the X chromosome in both humans and mice.

Together, these studies suggest that IL-13 may have a neuroprotective role in MS. Although this may be different in other neurodegenerative diseases that, unlike MS, are not characterized by a severely-compromised blood-brain barrier, and are not primarily mediated by peripheral immune cells, IL-13 and IL-4 also showed protection in a mouse model of Alzheimer’s disease (AD). Specifically, intracerebral injection of a mixture of IL-13 and IL-4 reduced amyloid deposition and improved spatial learning and memory in an AD transgenic mouse model when applied to young mice but did not show protective effects when administered in adult animals [79].

## 6. Parkinson’s Disease

The IL-13 system may have a specific role in the pathogenesis and/or the progression of Parkinson’s disease (PD). Data mining using the Online Mendelian Inheritance in Man (OMIM) database [80] showed that IL-13Rα1 lies within the PARK12 region of susceptibility to PD. Although PARK12 comprises a large portion of DNA, it is located on the X chromosome, an observation that may be of interest in that PD has a higher incidence in men than in women. Even more intriguing was the finding that expression of IL-13Rα1 in the brain appeared to be specific to the dopaminergic neurons of the VTA and of the SNc, the region affected by PD. Double-immunostaining studies also revealed that approximately 80% of the SNc neurons expressing the dopaminergic marker tyrosine hydroxylase also expressed IL-13Rα1 [1].

The possible contribution of IL-13Rα1 to neuronal fate was measured using a pro-inflammatory experimental mouse model of PD. Animals received periodic peripheral intraperitoneal injections of bacterial LPS over a period of six months, a regimen previously demonstrated to induce central loss of dopaminergic SNc neurons [81]. Comparative analysis showed that mice lacking IL-13Rα1 were protected from neuronal loss when compared to their wild-type littermates, suggesting a neurotoxic action of its ligands, IL-13 and/or IL-4. *In vitro* experiments using a dopaminergic cell line showed, however, that neither IL-13 nor IL-4 had cytotoxic effects when administered alone. However, both cytokines increased the toxicity of non-toxic doses of oxidants in a dose-dependent manner.

Thus, activation of IL-13Rα1 may be one of the mechanisms whereby the vulnerability of dopaminergic neurons is increased during inflammation, when both cytokines and ROS are produced. On the other hand, the lack of neurotoxicity of IL-13 or IL-4 in the absence of ROS suggests that these cytokines may be capable of regulating neuronal function by affecting the neurobiology of those neurons that participate in reward, addiction, and motor control.

Investigating these phenomena is likely to provide important information on the mechanisms of how IL-13 and IL-4 and, more generally, the immune system, may be capable of influencing behavior or can contribute to neurodegeneration.

## 7. Conclusions

Although in its infancy, the investigation of the central role of the interleukins 13 and 4 has is an exciting area of research. What makes it so attractive is that these two cytokines can be produced locally in the CNS and are active on both microglia and neuronal cells. Of special interest is the fact that they act through a common heterodimeric receptor that is expressed in dopaminergic neurons. Although these two Th2 cytokines are considered anti-inflammatory, studies conducted so far show that they can have cytotoxic effects on both glia and neurons. Interestingly these actions are not due to an intrinsic toxicity of IL-13 and IL-4 but rather to their ability to increase the cellular susceptibility to oxidative stress. This suggest that under pathological conditions, such as neuroinflammation when reactive oxygen species are produced, IL-13 and IL-4 can participate to tissue damage and thus to Parkinson’s disease or other neurodegenerative disorders. Instead, under physiological conditions, these two cytokines can contribute to the regulation of neuronal function via direct action through neuronal IL-13Rα1. Thus, they have the requisites of being potential neuromodulators.
